# Inflammation in Focus: The Beginning and the End

**DOI:** 10.3389/pore.2021.1610136

**Published:** 2022-01-04

**Authors:** Anna L. Kiss

**Affiliations:** Department of Anatomy, Histology and Embryology, Semmelweis University, Budapest, Hungary

**Keywords:** EMT, inflammatory cytokines, reactive oxygen species, receptor internalization, chronic inflammation

## Abstract

The inflammation is an important biological response induced by various harmful stimuli, like viruses, bacterial infections, toxins, toxic compounds, tissue injury. During inflammation inflammatory cytokines and reactive oxygen species are produced. Inflammatory cytokines act on various receptors present on the plasma membrane of target cells. To initiate signaling cascade, and activate transcription factors, receptors should be internalized and enter the early endosomes, where the members of the signaling cascade can meet. The further cytoplasmic fate of the receptor plays crucial role in the progression and the course of inflammation. Usually acute inflammation removes injurious stimuli and helps to regain the normal healthy status of the organism. In contrast to this the uncontrolled chronic inflammation—stimulating other than immune cells, inducing transdifferentiation—can provide base of various serious diseases. This paper draws the attention of the long-lasting consequence of chronic inflammation, pointing out that one of the most important step in medication is to identify in time the factors initiating and maintaining inflammation.

## Inflammation

The inflammation is a defense process of the body, a biological response of the immune system to harmful stimuli. The inflammation can be triggered by various pathogens (viruses, bacteria) toxins, toxic compounds, tissue injury [1]. These harmful stimuli initiate a chemical signaling cascade, activating leukocytes, that then produce and release inflammatory cytokines [2], such as interleukin-1β (IL-1β), interleukin-6, tumor necrosis factor-α (TNF-α). These cytokines interact with and activate receptors (IL-6R, TNFR-1, TNFR-2, TLR4, GM-CSFR etc.) [3]. Receptor activation triggers the phosphorylation of various signaling molecules such as mitogen-activated protein kinase (MAPK), nuclear factor kappa-B (NF-kB), Janus kinase (Jak), resulting in the activation of various transcription factors. This coordinate activation of signaling molecules regulates the level of inflammatory mediators in resident tissue cells, and recruit inflammatory cells from the blood [4,5]. Thus the acute inflammation is a protective mechanism, removes the injurious stimuli and initiates a healing process, restoring the homeostasis of the organism [6].

Uncontrolled acute inflammation, however, can become chronic, and can provide the base of a variety of serious, chronic diseases (tumors, a variety of neurogenerative diseases like Alzheimer disease, Parkinson disease, multiple sclerosis, lateral sclerosis, autoimmune diseases, diabetes, cardiovascular diseases, fibrosis etc.) [7–9]. Although the pathogenesis of these diseases is different, in most cases the inflammatory mediators, the regulatory and signaling pathways are common. In all cases 1) receptors must be present on the cell membrane and should be internalized (to register and transmit signals through the plasma membrane into the cell interior); 2) the members of the signaling cascade (various kinases, transcription factors) should meet in a platform called “signaling organelles/endosomes” [10], where they become activated; 3) activated transcription factors have to be translocated into the nucleus, to regulate inflammatory genes, resulting in inflammatory cytokine synthesis and release into the environment.

## Reactive Oxygen Species and Inflammation

Reactive oxygen species (ROS) are side-products of normal cell metabolism and produced in various cellular compartments like endoplasmic reticulum, mitochondria, peroxisomes. These reduced metabolites, with their strong oxidative capabilities, oxidize proteins, lipids, cellular constituents and can cause serious DNA damage. In physiological concentration ROS act as second messengers and function as signaling molecules in cell growth, cell adhesion and cell differentiation [11,12]. As second messengers, ROS posttranslationally modify proteins by oxidizing redox-sensitive cystein residues [13]. An increasing number of evidence show that reactive oxygen species are involved in initiation, progression and resolution of inflammatory responses. Chronic inflammation results in increasing ROS production. In turn, ROS regulate various types of kinases and transcription factors, such as nuclear kappa B (NF-kB), that are involved in the activation of pro-inflammatory genes [14,15]. Overproduction of ROS during chronic inflammation results in cell and tissue injury driving to serious diseases [13,16]. Neutrophil granulocytes are the primary ROS producers in the immune system, but other phagocytic cells, like macrophages, are also able to produce ROS significantly contributing to the increased ROS level. During post-bacterial phagocytosis NADPH oxidase (NOX) bound to the phagosomal membrane is activated and produces superoxide [17]. Altered metabolic activity, oxidative stress can also induce ROS production. Inflammatory cytokines activate STAT3, that is translocated into the nucleus, and acts as a transcription factor, regulating the transcription of inflammatory genes [18]. Recently it was shown, that a pool of Ser727 phosphorylated STAT3 translocates into the mitochondria where it stimulates ROS production [19–22], proving that inflammatory cytokines can directly stimulate ROS production. During chronic inflammation the ROS and inflammatory cytokine production are most probably interactive, orchestrated and synchronized and they magnify each other’s effect.

## Inflammation-Induced Epithelial-to-Mesenchymal Transition

**FIGURE 1:**
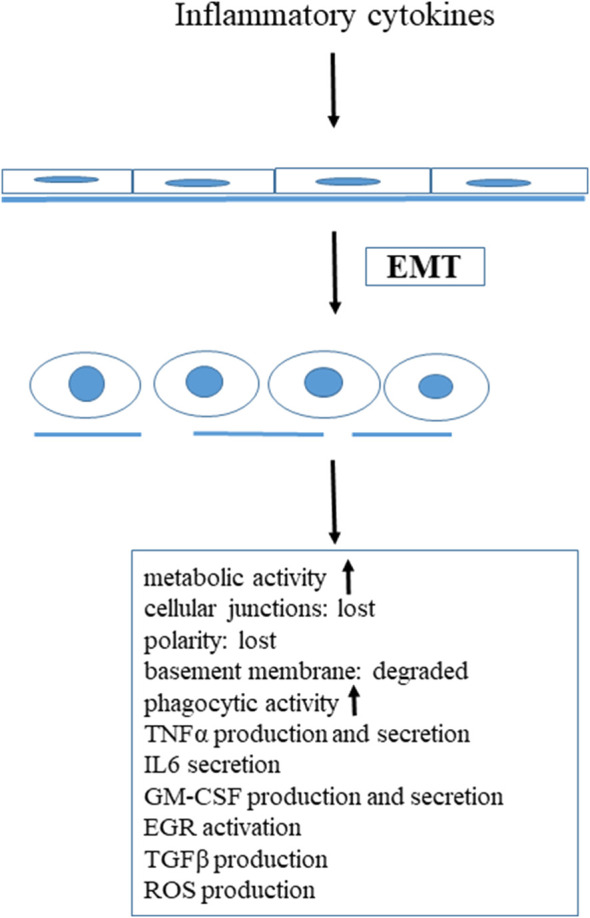
Inflammatory stimuli induced transdifferentiation of mesenteric mesothelial cells.

Nowadays it is well known that not only immune cells are able to respond to inflammatory stimuli and produce inflammatory cytokines. Under special circumstances, such as inflammatory stimuli, epithelial cells undergo a transition called epithelial-to-mesenchymal transition (EMT). EMT is an important biological process, triggered by various extracellular signals, including cytokines, growth factors and extracellular matrix components [23]. Three types of EMT are known, one of them, the EMT type II is associated with inflammation, wound healing, tissue regeneration and organ fibrosis [24]. It has been demonstrated that one of the most important factor that initiate EMT type II is the transforming growth factor (TGFβ) [25]. During this inflammation-induced EMT, epithelial cells undergo a complex proteomic remodelling, a series of morphological and biochemical changes [26]. In our *in vivo* system (rat mesenteric mesothelial cells) we found that inflammatory stimuli (Freund’s adjuvant induced peritonitis) led to prominent, spectacular morphological and biochemical changes in these epithelial cells. These mesothelial cells lost their polarity, E-cadherin and β-catenin synthesis was down-regulated, as a result of this cellular junctions were disassembled [23,27]. The basement membrane degraded, the cytoskeleton was remodelled and reorganized [27]. The volume of the cytoplasm, the number of the cytoplasmic organelles (rough ER, mitochondria, Golgi vesicles) dramatically increased, indicating that the metabolic rate and synthetic activities of these cells highly increased, but autophagy was blocked [28,29]. We have shown that healthy, non-treated mesothelial cells express toll-like receptor 4 (TLR4) as well (our unpublished results), that is responsible to recognize pathogens and participate in the activation of the inflammatory stimulus [9]. As a result of inflammation, mesothelial cells start to express macrophage markers (OX43, ED1) [30,31], synthesize and secrete pro-inflammatory cytokines, TNFα, IL-6 [32,33], their phagocytic activity is highly increased [32]. Inflammatory stimuli also initiate the expression and nuclear translocation of an unique transcription factor, EGR1 [32], which is known to affect the monocyte/macrophage lineage [34–37]. All these data provide strong evidence that under inflammatory stimuli, as a result of EMT, mesothelial cells transdifferentiate into macrophages or macrophage-like cells. Granulocyte-macrophage-colony stimulating factor (GM-CSF) was originally defined as a member of the hematopoietic cytokine family, promoting the survival and activation of granulocytes, macrophages and dendritic cells differentiation *in vivo* [32]. In our *in vitro* system (mesentery culture), GM-CSF treatment resulted in exactly the same morphological and biochemical changes as Freund’s adjuvant treatment proving that GM-CSF itself induced EMT, having an inflammatory effect as well [32]. GM-CSF-treated mesothelial cells synthesize and release GM-CSF and express GM-CSF receptor (both α and β) as well, constituting an autocrine, self-stimulating system. These data strongly support the previous observations, that GM-CSF can be locally produced [38–40], and point out the importance of GM-CSF in promoting inflammation. Taking together all these data, we can conclude that parallel to the morphological alterations, the inflammatory cytokines activated epithelial cells (in our case mesothelial cells) themselves started to produce pro-inflammatory cytokines, expressed cytokine receptors on their plasma membrane constituting an autocrine, self-stimulating, self-generating system. Thus these cells can contribute, actively take part in the inflammatory response (Figure 1).

## Receptors: Key Players in Response to Inflammatory Stimuli

**FIGURE 2:**
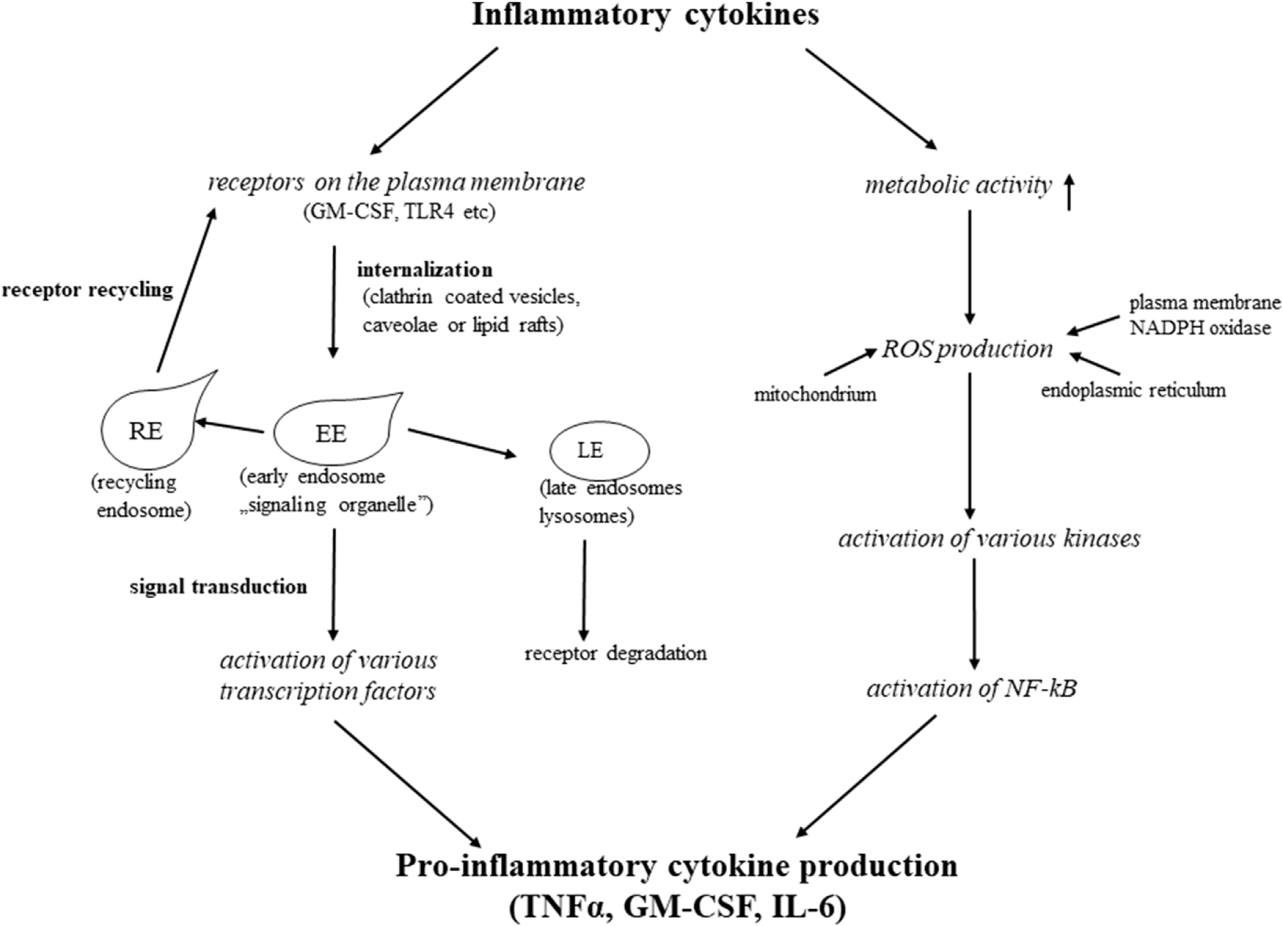
Mechanisms of inflammatory cytokines actions.

Extracellular stimuli inducing inflammatory response are hydrophilic molecules that require specific receptors on the plasma membrane of the target cell to transmit the signal into the cell interior where further steps of the signal transduction (transcription factors activation) take place. Thus membrane receptors are key elements of the inflammatory response, their presence on the plasma membrane and their intracellular fate determine how far the inflammatory response lasts, and how strong it is. Most of the cytokine receptors consist of several chains associated with different non-receptor kinases. Ligand binding to the receptors (TLR4, GM-CSFR, TNFR-1, and 2 etc.) induces synchronized and orchestrated phosphorylation events, initiating a signaling cascade (MAPK, NF-kB, JAK, STAT pathways) in the cell [41–44]. For the interaction between downstream molecules and initiating efficient signaling, membrane receptors, with their cargo, have to be internalized *via* clathrin-coated vesicles, caveolae (highly hydrophobic caveolin-1 containing plasma membrane invaginations), or lipid rafts [45,46]. After pinching off these vesicles from the plasma membrane, they fuse with early endosomes or “signaling” endosomes, (also called signaling organelles) [45,47–49]. Early or “signaling” endosomes provide a platform, a “meeting room” for the interactions with the down-stream elements of various signaling cascades (“signaling crosstalk”) [10]. After initiating signal, the intracellular route of the receptor and its fate plays a crucial role in the inflammatory response. If the receptor recycles to the cell surface, another signaling cycle can start, and the inflammatory stimulus is being continuously transmitted to the cell interior. Alternatively, if the cell directs the receptor to the degradative (lysosomal) pathway, the inflammation is stopped, and the cell can start to regenerate itself. In our experimental system we have shown that for the efficient signal transduction the receptor internalization is essential. When blocking the caveola-mediated internalization of GM-CSF receptor β by dynasore, (a potent inhibitor of small GTP-ase, dynamin [50]), the downstream element of GM-CSF signaling cascade, the STAT5, was not phosphorylated [46]. We also found that at the initial period of inflammation the receptor was present in early endosomes, where the STAT5 phosphorylation most probably occurs. While the inflammatory reaction was in course, the receptor was detected in recycling endosomes. When mesenteric mesothelial cells started to regenerate, the receptor was found in Rab7 positive late endosomes, and directed to a degradative/lysosomal pathway [46]. These results clearly show that removing the receptors from the cell surface, the inflammation stops and the regeneration can start, proving that the intracellular fate of the receptors should crucially determine the course of the inflammation (Figure 2).

## Clinical Aspects

Nowadays it is more and more accepted that chronic inflammation provides the base of serious chronic, non-inflammatory diseases including cardiovascular, neurodegenerative and bowel diseases, diabetes, arthritis, fibrosis and cancer. In the aetiology of the Alzheimer’s disease scientists found links between various pathogens and the development of the disease [51–53]. There are data indicating that in lateral sclerosis and in multiple sclerosis bacterial or viral infection can be one of the factors that initiates the disease. In normal healthy circumstances the inflammatory mediators are expressed at a low level and have only a few effects on the central nervous system. Neurons, astrocytes, microglial cells and oligodendrocytes can produce inflammatory mediators, and cytokine receptors are also expressed in the central nervous system [54]. Most probably these cytokines and receptors contribute to the normal physiological function of the CNS [55]. In pathological conditions, however, bacterial, viral, and fungi infections as well as tissue injuries, the pro-inflammatory cytokine level is highly elevated, partly because they are produced by the microglial cells and the recruited macrophages at the site of injury. Cytokines can also be produced at the periphery, at sites of infections or injuries, and transported by the blood. They can cross the blood-brain barrier by active transport through the leaky region of the vascular endothelial layer. These blood-derived cytokines can significantly contribute to the increased level of inflammatory mediators in the CNS. Various forms of IL-1, TNFα, IL-6, together with free radicals, ROS (causing damage of lipids, proteins and DNA) can significantly promote myelin sheath loss, injury of neurons, inducing neurodegeneration, serious neurodegenerative diseases like Alzheimer’s disease, lateral sclerosis, multiple sclerosis, Parkinson disease [56].

Chronic inflammation often results in fibrosis of various organs. Following injury, fibrosis plays an essential role in tissue repair. The aim of this process is to produce and deposit connective tissue components (fibers, and other extracellular matrix components), to regenerate the original tissue architecture [57]. Myofibroblasts are the main connective tissue components producing cells. Under inflammatory stimuli they derive from fibroblasts, macrophages, endothelial cells and many other cells types as well [57]. Injury results in local inflammation, and the inflammatory cytokines stimulate the transformation of the above mentioned cells into myofibroblasts. The long-lasting inflammatory stimuli, the uncontrolled chronic inflammation, however, can magnify the amplitudo of the production and deposition of the connective tissue components, resulting in chronic fibrosis in various organs (heart, kidney, liver etc).

Inflammation is far more complex process than it was thought originally. Under inflammatory stimuli (pathogens, toxins, tissue injuries etc.) inflammatory cytokines and ROS are produced primarily by immune cells and are transported by the blood circulation transporting them to everywhere in the body. Thus they act not only locally, instead their effect is rather systemic. An increasing number of evidence support that other cells, including epithelial cells, can respond to inflammatory stimuli, and can contribute to chronic inflammation. As it was discussed before mesenteric mesothelial cells are also equipped, or under inflammatory stimuli, can be equipped with all the tools necessary for inflammatory responses [28,29,31–33]. Under inflammatory stimuli they undergo transdifferentiation and as a result of this transformation they become able to produce inflammatory cytokines and ROS. With this capability they join to the immune defend “army” of the body and amplify, multiply and enhance the inflammatory response.

New data are making our knowledge deeper and wider and the “story” is becoming more and more complex. Recently it has been demonstrated that obesity is also associated with chronic systemic inflammation and is a serious risk factor for development of breast, ovarian and endometrial cancers [57–59]. Adipocytes produce adipokines and steroid hormones, like estrogen. Obesity alters the production of these molecules resulting in an inflammatory microenvironment in which pro-inflammatory macrophages can produce inflammatory cytokines. Additionally, free fatty acids are released from the adipocytes in obese individuals stimulating toll-like receptor 4 (TLR4) and activating NF-kB signaling pathway. The latter upregulates secretion of TNFα, IL-6, IL-8, IL-1β creating a positive feed-forward loop to sustain chronic inflammation and cancer progression [60–63].

Recently the so-called post-Covid diseases draw the attention to the generalized effect of inflammation. During SARS-COV-2 virus infection not only the immune cells but—as a result of inflammation induced EMT—epithelial cells in the lung (alveolar pneumocytes, endothelial cells and pleural mesothelial cells) can also produce high amounts of cytokines, resulting in a “cytokine storm.” Since inflammatory cytokines induce EMT, the transdifferentiation of various cells (including epithelial cells present in the blood vessels, in the lung alveoli and in the pleura) into myofibroblast can easily occur. Myofibroblasts then produce large amount of connective tissue fibers and other components of the extracellular matrix, locally resulting in lung fibrosis [64]. If we consider that the inflammatory cytokines are transported by the blood everywhere in the body, they can affect all the organs of the body, and depending on the individual’s sensitivity, they can induce various chronic diseases, causing cardiovascular, neural and mental symptoms.

## Conclusion

Since chronic inflammation can provide the base of many serious, fatal diseases, in the preventive therapy to identify pathogens, viruses, bacteria inducing inflammation is crucial. Because of the long-lasting consequence of chronic inflammation (inducing serious diseases) the most critical steps in medication are to identify, recognize inflammation in time, to determine the factors (bacteria, viruses, tissue injuries) that are behind the inflammation and use the proper treatment. The treatment should be combined using antibiotics, anti-inflammatory and anti-oxidant drugs.
